# Safety, Tolerability, Pharmacokinetics, and Pharmacodynamics of the CGRP Binding Monoclonal Antibody LY2951742 (Galcanezumab) in Healthy Volunteers

**DOI:** 10.3389/fphar.2017.00740

**Published:** 2017-10-17

**Authors:** David Monteith, Emily C. Collins, Corinne Vandermeulen, Anne Van Hecken, Eyas Raddad, Joel C. Scherer, David Grayzel, Thomas J. Schuetz, Jan de Hoon

**Affiliations:** ^1^Eli Lilly and Company, Indianapolis, IN, United States; ^2^Omeros Corporation, Seattle, WA, United States; ^3^Center for Clinical Pharmacology, University Hospitals of Leuven, KU Leuven, Leuven, Belgium; ^4^Arteaus Therapeutics, LLC, Cambridge, MA, United States

**Keywords:** CGRP, dermal blood flow, humanized monoclonal antibody, headache, migraine disorders

## Abstract

**Background:** Calcitonin gene-related peptide (CGRP) is pivotal in the pathophysiology of migraine headaches and represents a promising target for migraine treatment. The humanized monoclonal antibody galcanezumab (LY2951742) binds to CGRP and may be effective in migraine prophylaxis.

**Objectives:** The primary objective was to evaluate the safety and tolerability of single and multiple doses of galcanezumab in humans. Secondary objectives included assessing the pharmacokinetics and evaluating target engagement.

**Methods:** A double-blind, randomized, placebo-controlled study (NCT 01337596) with single escalating and multiple subcutaneous (SC) doses of galcanezumab was performed in healthy male volunteers. Single doses of 1, 5, 25, 75, 200, and 600 mg of galcanezumab (*n* = 7/dose) or placebo (*n* = 2/dose) were injected SC in six consecutive cohorts of nine subjects each. One cohort of nine subjects received multiple (4) 150 mg doses of galcanezumab or placebo every other week. Target engagement was evaluated by measuring inhibition of capsaicin-induced increase in dermal blood flow (DBF).

**Findings:** Sixty-three subjects were randomized and included in the safety analyses. Galcanezumab was well tolerated in single doses (1–600 mg SC) and consecutive doses (150 mg SC). There was no dose-dependent difference in type or frequency of treatment-emergent adverse events, and no clinically meaningful difference when compared with placebo. Pharmacokinetics were linear. Galcanezumab induced a robust, dose-dependent, and durable inhibition of capsaicin-induced increase in DBF, supporting the continued clinical development of galcanezumab for prophylaxis in migraine patients.

## Introduction

Migraine ranks seventh among disabling disorders and third in prevalence, accounting for more than half of disabilities attributable to all neurological diseases (Murray et al., 2012). Episodic migraine associated with increased frequency of attacks can progress to chronic migraine (Bigal and Lipton, 2006, 2008; Lipton, 2011; Diener et al., 2012). Therefore, interruption of episodic migraine can block its progression to a chronic state.

Calcitonin gene-related peptide (CGRP) is a potent vasodilatory neuropeptide widely expressed in peripheral and central neurons (Lennerz et al., 2008; Eftekhari and Edvinsson, 2011; Warfvinge and Edvinsson, 2013). Considerable evidence implicates CGRP in the pathophysiology of migraine (Lassen et al., 2002; Ho et al., 2010; Geppetti et al., 2012; Karsan and Goadsby, 2015). Intravenous infusion of CGRP precipitated migraine headache in migraine patients, but not in normal volunteers (Lassen et al., 2002; Hansen et al., 2010), and CGRP blood levels were elevated in patients during spontaneous or nitroglycerin-induced migraine attacks (Goadsby et al., 1988, 1990; Juhasz et al., 2003; Vanmolkot et al., 2006). Sumatriptan reduced both migraine pain and CGRP blood levels (Juhasz et al., 2005; Vanmolkot et al., 2006). Imaging studies showed vasodilation of extracranial, but not intracranial, arteries ipsilateral to headache during a migraine attack, which were alleviated by sumatriptan (Asghar et al., 2011; Geppetti et al., 2012).

Several recent randomized clinical trials with small-molecule CGRP receptor antagonists, particularly olcegepant and telcagepant, showed that they effectively abort acute episodic migraine attacks (Olesen et al., 2004; Ho et al., 2008). Olcegepant was limited because it could only be formulated and administered intravenously (Durham and Vause, 2010). Telcagepant produced pain-free headache responses in acute migraine and decreased headache frequency with daily administration (Ho et al., 2014). However, chronic telcagepant therapy was associated with hepatotoxicity concerns (Ho et al., 2014), most likely related to the chemical structure of the compound.

The humanized monoclonal antibodies were developed as an alternative strategy to small-molecule CGRP antagonists for migraine prophylaxis. Currently there are three monoclonal antibodies (LY2951742 or galcanezumab, TEV-48125 or fremanezumab, and ALD-403 or eptinezumab) that bind to CGRP and 1 (AMG 334 or erenumab) that binds directly to the CGRP receptor in clinical development (Dodick et al., 2014a,b; Bigal et al., 2015a,b; Sun et al., 2016). All have proven successful in reducing the frequency of migraine headaches in early clinical trials as a preventive therapeutic (Dodick et al., 2014a,b; Bigal et al., 2015a,b; Sun et al., 2016). Previous studies with small molecule inhibitors of the CGRP receptor similarly demonstrate this inhibition is associated with preventing acute attacks (Olesen et al., 2004; Ho et al., 2008), as well as prevention of attacks (Ho et al., 2014).

Galcanezumab avidly binds to the human CGRP, with a binding affinity (K_D_) of 31 pM (4.5 ng/mL). Galcanezumab prevented the CGRP-dependent capsaicin-induced increase in dermal blood flow (DBF) in non-clinical studies with rats and non-human primates (Vermeersch et al., 2015). Moreover, galcanezumab inhibited capsaicin-induced vasodilation in non-human primates for 28 days after single administration, suggesting a long half-life, making galcanezumab a potentially valuable therapeutic agent in the prophylaxis of migraine headache (Vermeersch et al., 2015).

The present report is derived from the first in human dose Phase I study (NCT 01337596) and presents safety, tolerability, immunogenicity, and pharmacokinetic (PK) results obtained with single and multiple subcutaneous injections of up to 600 mg of galcanezumab to healthy male subjects. Additionally, the study investigated the inhibition by galcanezumab of capsaicin-induced DBF, which is a validated and well-established method for evaluating therapeutics targeting the CGRP pathway (Buntinx et al., 2015). These data guided the modeling of doses for further clinical trials. Finally, the influence of galcanezumab on vascular tone was evaluated as an exploratory biomarker. Although earlier non-clinical studies and clinical experience with small molecule inhibitors did not suggest a problem (Iovino et al., 2004; Olesen et al., 2004; Petersen et al., 2005a; Ho et al., 2008, 2014), the potential vascular effects of a therapeutic antibody with prolonged pharmacologic activity are unknown.

## Materials and methods

### Study design

**Figure 1:**
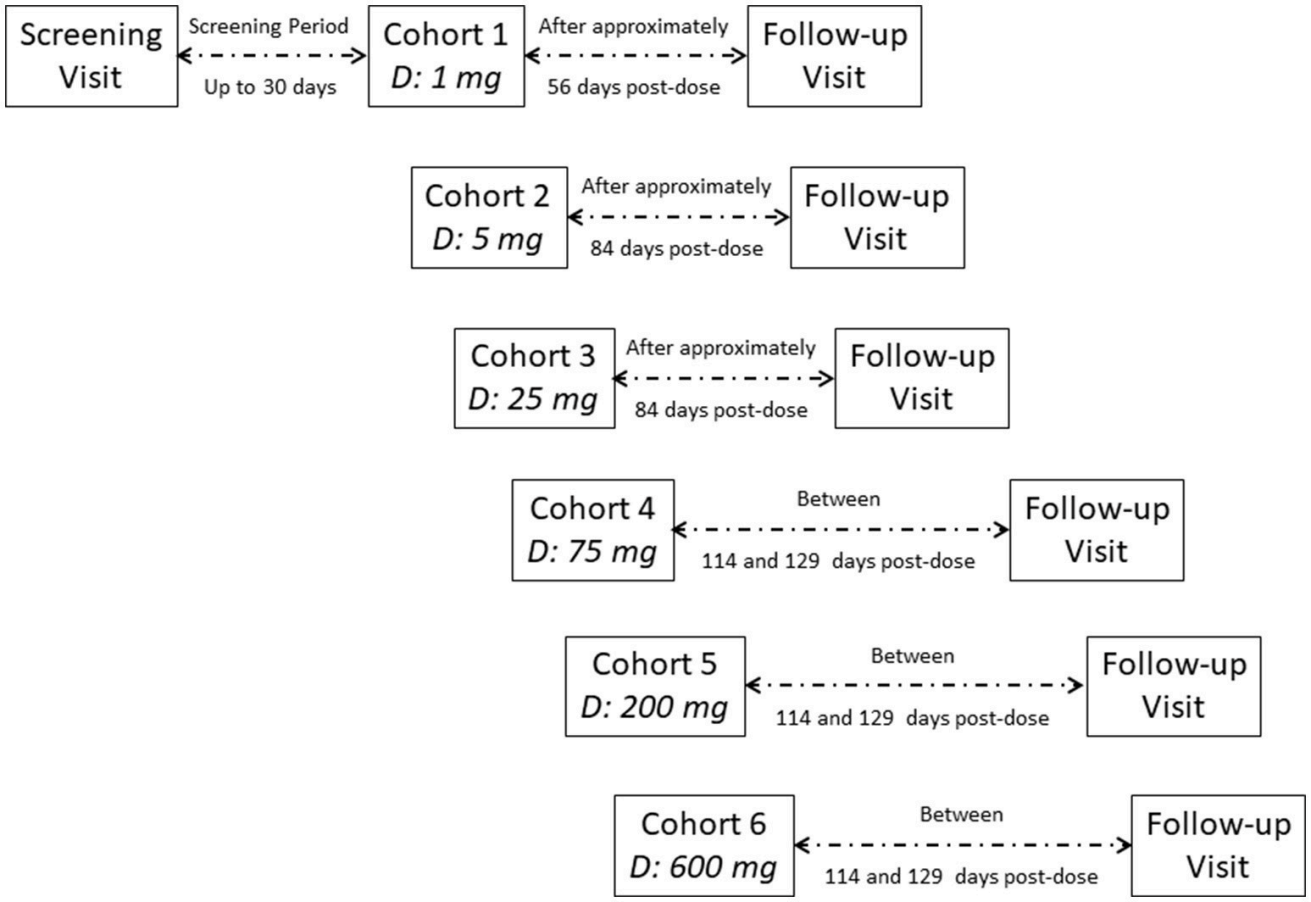
Disposition of subjects (enrolled subjects).

This was a single-center, double-blind, randomized, placebo-controlled study conducted at the Center for Clinical Pharmacology of the University Hospitals in Leuven, Belgium (NCT 01337596). The study involved two sequential parts: a single-ascending-dose (SAD) phase of six cohorts followed by one multiple-dose cohort. Figure 1 includes detailed information regarding trial conduct and subject randomization for the SAD part of the study.

In the SAD phase of the study, six cohorts of nine healthy male subjects each were dosed to assess six dose levels of galcanezumab administered subcutaneously (SC) in the abdominal wall at doses of 1, 5, 25, 75, 200, and 600 mg (Figure 1). Cohorts were designed with a sequential single-dose escalation scheme; within each cohort, seven subjects received galcanezumab and two received placebo. At each dose level, the first two subjects were dosed with one subject receiving a single SC dose of galcanezumab and the other receiving placebo (i.e., sentinel dosing). After an interval of at least 48 h and following review of all available safety data [i.e., adverse events (AEs), electrocardiograms (ECGs), vital signs, and laboratory parameters] of these two sentinel subjects, the seven remaining subjects were dosed all together. After an interval of at least 8 days and review of all safety data from all subjects within each cohort, the next cohort could be dosed with an increased dose. Subjects were admitted to the research unit on Day-1, received galcanezumab or placebo SC in the morning of Day-1, and were discharged on Day 3. Subsequently, they returned for outpatient visits on Days 5, 8, 14, 28, and 42. For Cohort 1 subjects (i.e., 1 mg), the final follow-up visit took place 56 days (±2 days) after dosing; for the remaining cohorts, the final follow-up visit took place 84–129 days after dosing, based on emerging PK data.

The multiple-dose cohort started after review of the safety and tolerability data from the 600 mg single-dose cohort (Figure 1). In the multiple-dose cohort, nine healthy male subjects were included to receive a total dose of 600 mg galcanezumab (*n* = 7) or placebo (*n* = 2). Subjects received one SC injection of 150 mg galcanezumab or placebo on Days 1, 15, 29, and 43 in an alternating site in the abdominal wall (1 injection site per day with 4 days of injections). The dose of 150 mg was chosen for two reasons. First, the dose of 150 mg was the maximal dose that could be administered as a single injection, and therefore, considered likely a maximum practical dose for assessing the efficacy in subsequent trials. Further, 150 mg as four injections did not exceed the maximum dose evaluated as a single dose. For their first dose, subjects were admitted to the research unit the evening before dosing (i.e., Day-1); for the remaining doses, subjects came to the unit on the morning of dosing. Subjects were discharged approximately 4 h after dosing and returned on an outpatient basis for scheduled study procedures. The final follow-up visit of subjects in the multiple-dose cohort was conducted approximately 4 months after the last dose.

### Study participants

Participants were healthy White males aged between 18 and 55 years inclusive, with a body mass index (BMI) ≥19.0 kg/m^2^. After having given written informed consent to participate in the study, all subjects followed the screening procedures within 30 days prior to dosing. Each subject participated in only one cohort of treatment.

The study was conducted following approval by the independent Ethics Committee of the University Hospitals of Leuven and in accordance with the Declaration of Helsinki, the International Conference on Harmonization Good Clinical Practice guidelines, and local regulations.

### Study assessments

#### Safety and tolerability

Subject safety was evaluated on the basis of reported AEs, physical examination, vital signs (i.e., diastolic blood pressure, systolic blood pressure, heart rate, and body temperature), ECGs, clinical chemistry, clinical hematology, and urinalysis. Tolerability was evaluated by AE reporting. The incidence of AEs was tabulated using classifications and terms from the Medical Dictionary for Regulatory Activities (MedDRA; version 14.0). Safety and tolerability data were collected on an ongoing basis.

#### Pharmacokinetics

After dosing, serial blood samples were collected for the determination of serum concentrations and PK parameters of galcanezumab. Concentration time profiles for galcanezumab were analyzed using standard non-compartmental methods of analysis. Area under the curve (AUC) and peak serum concentration (C_max_) were the primary PK parameters. Geometric means were estimated, and mixed-effect models were used to investigate dose proportionality. The apparent elimination half-life (*t*_1/2_) is also reported.

Enzyme-linked immunosorbent assay (ELISA) method was used for determination of serum levels of galcanezumab.

#### Pharmacodynamics

The secondary pharmacodynamic (PD) parameters included: (1) prevention of DBF changes induced by capsaicin as assessed by laser Doppler imaging (LDI) with the use of a Doppler perfusion imager (HR-LDPI system, Periscan PIMII; Perimed, Sweden) and (2) pulse wave analysis (PWA) for evaluating effects on vascular tone.

Target engagement (i.e., CGRP binding by galcanezumab) was evaluated by measuring the inhibition of capsaicin-induced changes in DBF by galcanezumab. Assessments of DBF were conducted 48–56 h following dosing and on the days indicated in **Figures 3**, **4**. The methodology is an established target engagement biomarker for evaluating CGRP-receptor antagonists in humans (Van der Schueren et al., 2008; Sinclair et al., 2010; Buntinx et al., 2015) as well as in monkeys (Vermeersch et al., 2015).

During the single-dose escalation phase only, the potential effect of galcanezumab on arterial stiffness (i.e., vascular tone) was evaluated as an exploratory endpoint. The aortic augmentation index (AIx@HR75) was used as a measure of arterial stiffness and derived from radial artery pressure waveforms. Radial artery pressure waveforms were measured non-invasively based on tonometry evaluation of the radial artery as previously reported (Vanmolkot and de Hoon, 2006; Van der Schueren et al., 2007, 2011). These evaluations were performed at screening (predose/baseline) and on Days 3, 14, 28, and 42.

Antidrug antibodies (ADA) were determined in serum from patients by an ELISA assay which determined galcanezumab-reactive binding proteins after acidification and release of any bound drug and detection with biotin-labeled galcanezumab. The assay was validated to be drug tolerant at galcanezumab concentrations up to 20 μg/mL. An initial screening assay was performed to determine the presence of ADA and then samples were assayed to determine the titer of ADA. Subsequent, effects of the presence of ADAs were determined by comparison of PK and PD in subjects with and without ADA titers.

### Statistics

The sample size (seven active and two placebo per cohort) was determined to be adequate for Phase I studies evaluating safety and PK parameters and was not powered on the basis of inferential statistical hypothesis testing. For tables and graphs, all data from subjects receiving placebo from each cohort are combined.

Subjects were randomly assigned to galcanezumab or placebo treatment groups. To that end a computerized randomization table was prepared by an external statistician and provided to the study site unblinded pharmacist.

Laboratory parameters, ECGs, and vital signs were summarized descriptively for each regimen. Laboratory values and vital signs results were listed. Safety analyses were conducted for all enrolled subjects who received a dose of study drug, whether or not they completed all protocol requirements. DBF and PWA results were listed and summarized using standard descriptive statistics.

## Results

Sixty-three subjects were randomized to study treatments. During the single-dose-escalation phase, 52 of 54 subjects completed the study as per protocol. Two subjects received a single dose of 200 mg galcanezumab but withdrew their consent for personal reasons prior to completing the final follow-up visit. As a result, 52 of the 54 subjects randomized in the single-dose-escalation phase completed the study. During the multiple-dose part of the study, all nine subjects completed the study as per protocol. From study drug administration to final follow-up visit, the study duration for the subjects in the single-dose-escalation phase varied from 54 to 161 days depending on the dose received. From study drug administration to final follow-up visit, the study duration for the subjects in the multiple-dose phase varied from 176 to 181 days. Demographic and baseline characteristics are presented in Tables 1, 2.

**Table 1:** Summary of demographic and baseline characteristics of single-dose-escalation phase.

**Dose, mg**	**No**.	**Age, Year**	**Weight, kg**	**Height, cm**	**BMI, kg/m^2^**
Placebo	12	30.8 (21–45)	77.73 (66.2–89)	179.3 (163–194)	24.28 (19.8–30.2)
1	7	27.6 (19–46)	75.66 (65.4–88.4)	178.7 (169–190)	23.84 (19.5–29.2)
5	7	29 (19–40)	80.6 (62.6–94.4)	179.3 (167–185)	25.04 (19.8–27.9)
25	7	31.9 (19–54)	75.69 (56.8–87.6)	180.9 (168–187)	23.06 (20.1–25.1)
75	7	30.9 (21–45)	79.03 (71.4–89.2)	180.7 (173–188)	24.27 (20.2–26.9)
200	7	37 (23–54)	91.94 (77.8–108.2)	179.7 (172–186)	28.54 (23.2–34.2)
600	7	33.9 (18–52)	82.8 (65.8–102.2)	178.3 (174–188)	26.04 (21.2–33.8)
Overall	54	31.5 (18–54)	80.24 (56.8–108.2)	179.5 (163–194)	24.94 (19.5–34.2)

*Data are presented as mean (range). BMI, body mass index*.

**Table 2:** Summary of demographic and baseline characteristics of multiple-dose phase.

**Parameter**	**Placebo (*n* = 2)**	**Galcanezumab 600 mg (*n* = 7)**	**Overall (*N* = 9)**
Age (year)	22.5 (22–23)	22.4 (21–26)	22.4 (21–26)
Weight (kg)	75.5 (74.2–76.8)	66.4 (51.6–88.8)	68.42 (51.6–88.8)
Height (cm)	182 (179–185)	173.6 (161–186)	175.4 (161–186)
BMI (kg/m^2^)	22.8 (22.4–23.2)	21.96 (18.5–27.4)	22.14 (18.5–27.4)

*Data are presented as mean (range). BMI, body mass index*.

All 63 subjects were included in the safety analyses. All subjects who received galcanezumab (42 in the single-dose-escalation phase and seven in the multiple-dose phase) were included in the PK and PD analyses.

### Safety

Galcanezumab was well tolerated in the 1–600 mg dose range when administered as a single subcutaneous dose and after four consecutive doses of 150 mg administered over 6 weeks. AEs are summarized in Tables 3, 4 for single dose and multiple dose, respectively. AEs were transient with no apparent relationship with the prolonged systemic drug exposure (indicated by the long half-life of galcanezumab). In subjects receiving galcanezumab, the most common AEs were headache, nasopharyngitis, hematuria, and contact dermatitis; however, with the exception of hematuria which was not present in placebo, the frequencies of these events were similar to placebo. Other frequently reported AEs in subjects receiving galcanezumab were diarrhea, toothache, and increased alanine aminotransferase (ALT). There were no clinically significant changes from baseline in safety measures in subjects who received galcanezumab. There were no apparent differences between galcanezumab dose groups or between galcanezumab dose groups and placebo in terms of the frequency of any AEs or changes from baseline in vital signs, laboratory values, or ECG parameters.

**Table 3:** Treatment-emergent adverse events experienced by at least one galcanezumab-treated subject after single-dose administration by dose cohort and MedDRA preferred term.

**MedDRA preferred term**	**Galcanezumab dose cohort, mg**							**Placebo (*n* = 12)**
	**1 (*n* = 7)**	**5 (*n* = 7)**	**25 (*n* = 7)**	**75 (*n* = 7)**	**200 (*n* = 7)**	**600 (*n* = 7)**	**Total (*n* = 42)**	
At least two TEAEs	6 (86)	5 (71)	7 (100)	4 (57)	7 (100)	5 (71)	34 (81)	9 (75)
Headache	1 (14)	3 (43)	2 (29)	0 (0)	4 (57)	2 (29)	12 (29)	5 (42)
Nasopharyngitis	3 (43)	1 (14)	0 (0)	0 (0)	1 (14)	1 (14)	6 (14)	4 (33)
Dermatitis contact	1 (14)	0 (0)	4 (57)	0 (0)	0 (0)	0 (0)	5 (12)	1 (8)
Diarrhea	0 (0)	0 (0)	1 (14)	1 (14)	1 (14)	1 (14)	4 (10)	0 (0)
Toothache	0 (0)	1 (14)	1 (14)	0 (0)	0 (0)	1 (14)	3 (7)	0 (0)
ALT increased	0 (0)	0 (0)	1 (14)	0 (0)	1 (14)	1 (14)	3 (7)	0 (0)
Hematuria	0 (0)	0 (0)	1 (14)	0 (0)	1 (14)	1 (14)	3 (7)	0 (0)
Dental caries	0 (0)	0 (0)	1 (14)	0 (0)	0 (0)	1 (14)	2 (5)	0 (0)
Vomiting	0 (0)	1 (14)	0 (0)	0 (0)	0 (0)	1 (14)	2 (5)	1 (8)
Injection site hemorrhage	0 (0)	0 (0)	0 (0)	1 (14)	1 (14)	0 (0)	2 (5)	0 (0)
Gastroenteritis	0 (0)	0 (0)	0 (0)	1 (14)	1 (14)	0 (0)	2 (5)	0 (0)
Upper respiratory tract infection	0 (0)	0 (0)	1 (14)	0 (0)	0 (0)	1 (14)	2 (5)	1 (8)
AST increased	0 (0)	1 (14)	1 (14)	0 (0)	0 (0)	0 (0)	2 (5)	0 (0)
Blood CPK increased	0 (0)	1 (14)	1 (14)	0 (0)	0 (0)	0 (0)	2 (5)	0 (0)
Back pain	0 (0)	1 (14)	0 (0)	1 (14)	0 (0)	0 (0)	2 (5)	1 (8)
Leukocyturia	1 (14)	1 (14)	0 (0)	0 (0)	0 (0)	0 (0)	2 (5)	0 (0)
Oropharyngeal pain	0 (0)	1 (14)	0 (0)	1 (14)	0 (0)	0 (0)	2 (5)	0 (0)
Erythema	0 (0)	2 (29)	0 (0)	0 (0)	0 (0)	0 (0)	2 (5)	0 (0)
Pain in extremity	0 (0)	1 (14)	0 (0)	0 (0)	1 (14)	0 (0)	2 (5)	0 (0)

*Data are presented as No. (%) of patients experiencing the TEAE. ALT, alanine aminotransferase; AST, aspartate aminotransferase; CPK, creatine phosphokinase; MedDRA, Medical Dictionary for Regulatory Activities; TEAE, treatment-emergent adverse event*.

**Table 4:** Treatment-emergent adverse events experienced by at least one galcanezumab-treated subject after multiple-dose administration by MedDRA preferred term.

**MedDRA preferred term**	**Galcanezumab 150 mg (*n* = 7)**	**Placebo (*n* = 2)**
Subjects with at least 1 TEAE	7 (100)	2 (100)
Nasopharyngitis	2 (29)	2 (100)
Injection site pain	2 (29)	0 (0)
Hematuria	1 (14)	1 (50)
Oral herpes	1 (14)	1 (50)
Arthropod bite	1 (14)	0 (0)
Chest discomfort	1 (14)	0 (0)
Dermatitis contact	1 (14)	0 (0)
Flatulence	1 (14)	0 (0)
Gastroenteritis	1 (14)	0 (0)
Influenza like illness	1 (14)	0 (0)
Injection site erythema	1 (14)	0 (0)
Mouth ulceration	1 (14)	0 (0)
Pain in extremity	1 (14)	0 (0)
Pharyngitis	1 (14)	0 (0)
Productive cough	1 (14)	0 (0)
Rash	1 (14)	0 (0)
Tension headache	1 (14)	0 (0)
Toothache	1 (14)	0 (0)
Vessel puncture site hematoma	1 (14)	0 (0)

*Data are presented as No. (%) of patients experiencing the TEAE. The following TEAEs occurred in placebo in only 1 (50%) subject each: bronchitis, headache, injection site hemorrhage, and nasal congestion. MedDRA, Medical Dictionary for Regulatory Activities; TEAE, treatment-emergent adverse event*.

Given the proposed mechanism of action of galcanezumab, AEs associated with cardiovascular function were of special interest. Few cardiovascular-related AEs were reported (one event each in two subjects receiving galcanezumab and one subject receiving placebo). In the two subjects who received galcanezumab, the events did not appear to be temporally associated with dosing of galcanezumab. Increased heart rate was reported by Subject 1507 (200 mg galcanezumab). This event occurred 28 days after galcanezumab administration, was mild in intensity, and was considered not related to galcanezumab. Subject 1607 experienced an increase in systolic blood pressure 84 days after receiving a single dose of 600 mg galcanezumab. The Investigator considered the event unlikely to be related to the study drug. Subject 1105, who received placebo, experienced an increase in orthostatic heart rate response. Other AEs that could be potentially related to cardiovascular function included postural dizziness (reported in one subject each receiving 1 mg galcanezumab and placebo) and presyncope (one subject receiving placebo). There are no consistent time- or dose-related effects to consider that galcanezumab was associated with any cardiovascular effects.

Of additional interest were any particular effects on liver function. During the single-dose escalation phase, it was noted that five subjects had transient elevations (i.e., more than two times the upper normal range) of the hepatic enzymes aspartate aminotransferase (AST) and ALT. Of these, two had participated in strenuous physical activity that likely contributed to the elevations; both were considered by the Investigator to be unrelated to galcanezumab. The other three subjects reported 2.7-, 6.5-, and 3.1-fold ALT increases more than 80 days after receiving a single dose of 5, 200, and 600 mg galcanezumab, respectively. Furthermore, the subject receiving 200 mg of galcanezumab and presenting with the 6.5-fold increase in ALT developed liver function abnormalities, consisting of elevated AST, ALT, alkaline phosphatase, and (total) bilirubin, typical for acute cholestatic hepatitis at 84 days after dosing with galcanezumab. Given the previously reported hepatotoxicity associated with the small-molecule CGRP-receptor antagonist telcagepant, an extensive workup was performed to identify the cause of the mixed picture of cholestatic and hepatocellular changes. It was concluded that the concomitant intake of ciprofloxacin, prescribed together with paracetamol and ibuprofen for the treatment of a urinary tract infection, was the most likely cause During the multiple-dose phase, all clinical laboratory parameters were within normal ranges, including liver function tests.

ADA was assessed predose and at 28 and 42 days after drug administration in the single dose study. Eleven (26%) of galcanezumab-treated subjects demonstrated treatment emergent-ADA (three of whom had pre-existing that increased in titer after dosing), with low titers between 1:10 and 1:80. These were 4, 5, 1, and 1 subjects at single doses of 5, 25, 75, and 600 mg, respectively. No dose response was demonstrated, and doses of 1 and 200 mg did not demonstrate detectable ADA. With repeat biweekly dosing at 150 mg dose, ADA was assessed predose and on day 50, 71, and 176. No ADA was detected on day 50 and 71, while TE-ADA was detectable in 4 (57%) of galcanezumab-treated subjects with a titer between 1:20 and 1:160 on day 172. Although limited to this small number of subjects, the presence of ADA had no obvious effect on PK (serum concentrations) or PD (inhibition of capsaicin-induced DBF) compared with subjects who had no detectable ADA titers (data shown for multiple-dose cohort, Table 5).

**Table 5:** The relationship between galcanezumab serum concentration, ADA titer and capsaicin-induced DBF upon four biweekly galcanezumab dose administration.

**Subject**	**ADA Titer**	**Serum Galcanezumab (ng/mL)**	**DBF Pre-capsaicin**	**DBF Post-capsaisin**	**Fold increase**
701	ND	1,765	0.48	2.32	4.8
703	1:20	2,338	0.64	2.54	4.0
704	1:40	4,012	0.65	1.00	1.5
705	1:40	1,935	0.75	2.13	2.8
706	ND	2,599	0.75	1.66	2.2
707	ND	2,145	0.59	1.35	2.3
709	1:160	2,795	0.91	2.91	3.2

*PK, ADA, and DBF measurements shown are on Day 176 from the beginning of dosing, 133 days after the last galcanezumab dose administration*.

*ADA, Anti-drug antibodies; DBF, dermal blood flow; ND, not detected; PK, pharmacokinetics*.

#### Pharmacokinetics

**Figure 2:**
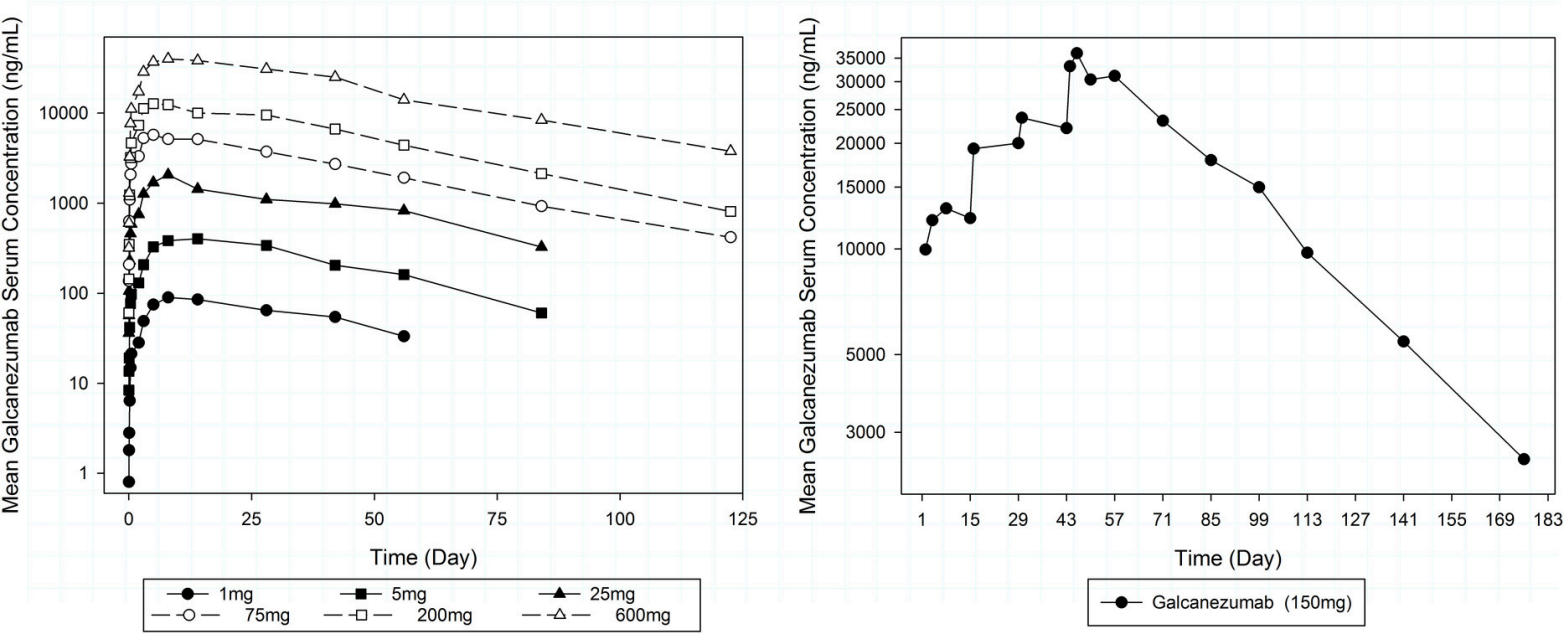
Mean serum galcanezumab concentration profiles over time by dose cohort after single **(left)** and multiple **(right)** biweekly dose administration.

Serum concentration-time curves of galcanezumab are provided in Figure 2. PK results following single-dose SC administration indicate that there was an extended period of absorption, with a median time to peak concentration (*T*_max_) between Day 7 and 14 (Table 6). C_max_ and the area under the concentration-time curve from dosing to infinity (AUC_(0−∞)_) were generally dose proportional over the full dose range (1 and 600 mg). The mean serum half-life (*t*_1/2_) of galcanezumab was similar at all dose levels at 25–30 days. PK results following four consecutive doses of 150 mg administered with a 14-day dosing interval were, for the most part, as predicted from single-dose administration. A 3.5-fold accumulation of drug concentrations was observed after the fourth dose but had not reached steady state. The median *T*_max_ was 3 days and was much shorter than the 7–14 day medians observed after single-dose administration. The geometric mean *t*_1/2_ was 31.9 days, which was slightly longer but consistent with the mean *t*_1/2_ observed after single doses.

**Table 6:** Mean (±SD) pharmacokinetic parameters of galcanezumab by dose cohort.

**Dose (mg)**	**Frequency**	***t*_1/2_, days**	***T*_max_, days^a^**	**C_max_, ng/mL**	**AUC, ng·day/mL^b^**
1	Single dose	27.7 (5.1)	13 (7–15)	97.46 (20.91)	4746 (562)
5	Single dose	25.1 (3.7)	14 (7–15)	425.8 (155.4)	20,510 (6,440)
25	Single dose	29.6 (5.7)	7 (4–39)	2,147 (843.8)	96,260 (29,140)
75	Single dose	28.2 (4.4)	7 (2–14)	6,449 (2,782)	280,900 (93,800)
200	Single dose	28.4 (7.1)	7 (4–13)	14,650 (5,366)	663,600 (242,900)
600	Single dose	30.3 (4.6)	7 (4–27)	45,990 (10 060)	2,290 000 (222,900)
150	Four biweekly doses^c^	32.0 (3.0)	3 (1–14)	37,210 (5,793)	1,959,000 (454,700)

*Cohort are the 7 subjects administered galcanezumab*.

a *Data are presented as median (range of values from individual subjects)*.

b *AUC(0–∞) for single dose, and AUC over the 2 week dosing period for multiple dose*.

c *Parameters are reported for pharmacokinetic profile after the fourth/last dose administration; note that steady state was yet to be achieved after the last dose*.

*AUC_(0−∞)inf_, area under the concentration-time curve from dosing to infinity; C_max_, peak serum concentration; t_1/2_, serum half-life; T_max_, time to peak concentration*.

#### Pharmacodynamics

**Figure 3:**
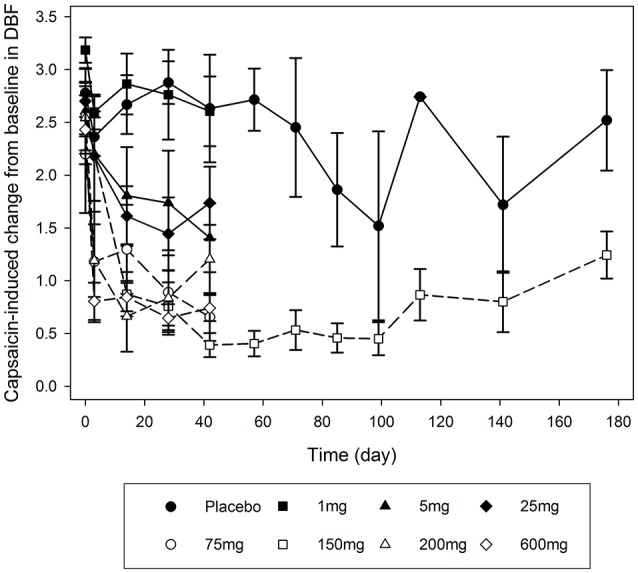
Mean profiles of the capsaicin-induced dermal blood flow changes over time (days after galcanezumab administration) for each single-dose and multiple-dose group. Dermal blood flow is presented as arbitrary perfusion units as determined by HR-LDPI system software (Periscan PIMII; Perimed, Sweden).

**Figure 4:**
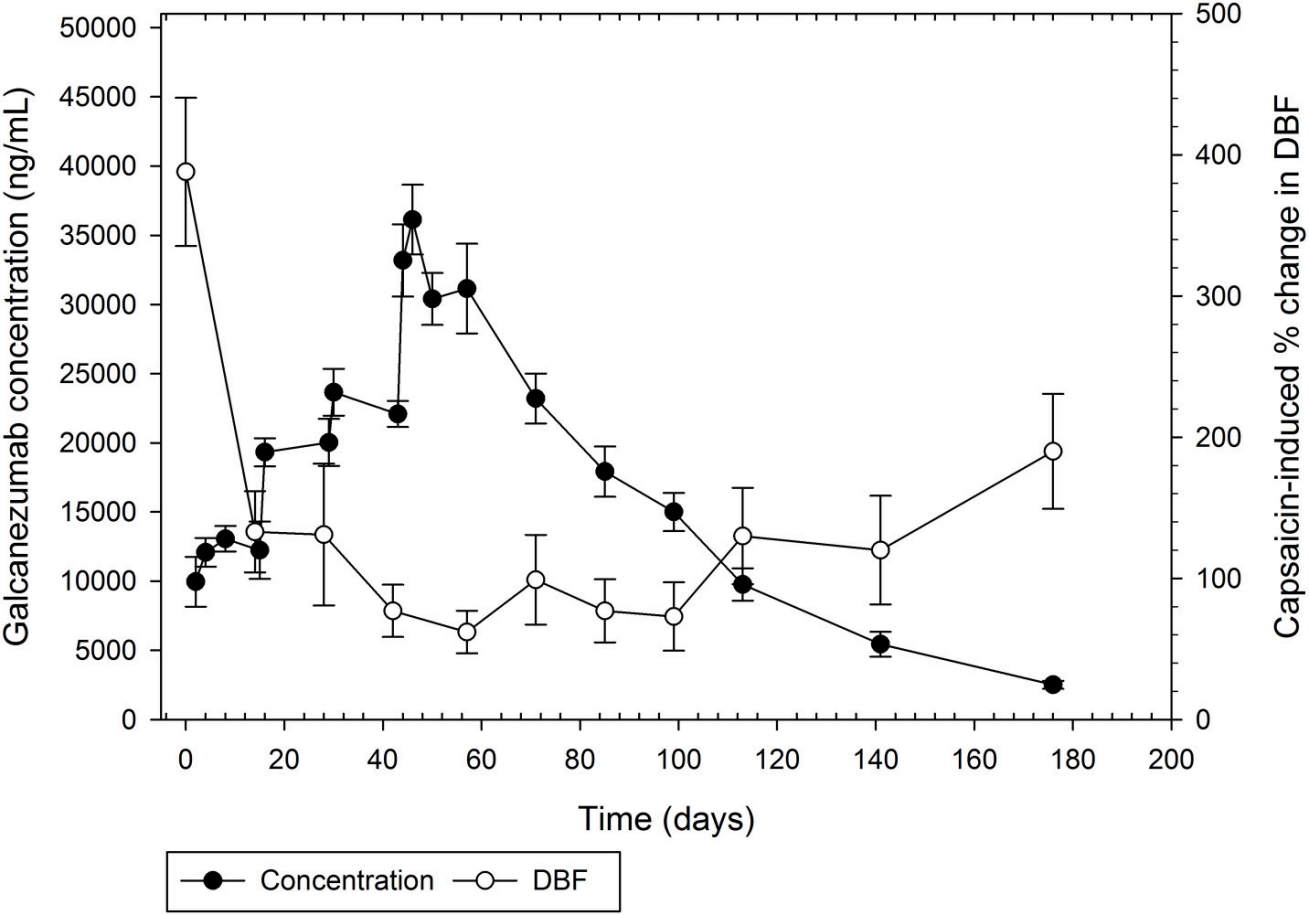
Mean serum concentrations of galcanezumab (±standard error of the mean) and fold change induction by capsaicin of dermal blood flow from baseline for repeat administration of 150 mg galcanezumab (Days 1, 14, 28, and 42).

Single-dose administration of galcanezumab demonstrated a dose-dependent inhibition of capsaicin-induced DBF starting at 5 mg between Day 28 and 42. At doses of 75, 200, and 600 mg, the effect was near maximal on Day 3, and remained maximal until Day 42, the last assessment following single-dose administration (Figure 3). After four consecutive doses of 150 mg, the induction of DBF by capsaicin was increasingly inhibited (Figure 4). The inhibition was sustained for up to 130 days after the last dose of galcanezumab. The maximum magnitude of the effect was similar to the effect of a single dose of 600 mg (Figure 3).

Further, galcanezumab demonstrates inhibition of capsaicin-induced DBF in a robust concentration-response relationship when compared with serum concentrations of galcanezumab (Figure 4). The extended period of inhibition is demonstrated as the serum concentrations steadily decrease; this result suggests that a concentration threshold for a maximal inhibitory effect is exceeded.

In the 1–600 mg dose range, single subcutaneous doses of galcanezumab did not influence the AIx@HR75 as a measure of arterial stiffness and wave reflection. Changes in AIx@HR75 did not appear to be significant on any day of assessment, at any dose. Additionally, on Day 14, estimates of the differences in least-squares means were not significant at any dose level (Table 7).

**Table 7:** Difference of AIx@HR75 between galcanezumab and placebo in change from predose by dose group on Day 14 after single subcutaneous injections to healthy subjects.

**Dose group**	**Mean change**	**95% CI**	***P*-value**
Galcanezumab 1 mg–placebo	−4.301	−13.394, 4.791	0.3445
Galcanezumab 5 mg–placebo	−2.051	−10.605, 6.502	0.6303
Galcanezumab 25 mg–placebo	−1.605	−10.718, 7.508	0.7235
Galcanezumab 75 mg–placebo	−0.807	−8.979, 7.366	0.8428
Galcanezumab 200 mg–placebo	1.301	−7.274, 9.875	0.7606
Galcanezumab 600 mg–placebo	−0.375	−8.531, 7.781	0.9264

*AIx@HR75, augmentation index at heart rate equal to 75; CI, confidence interval*.

## Discussion

This study primarily reports on the safety and tolerability of galcanezumab in healthy male subjects following single and repeated SC injections. Overall, galcanezumab was well tolerated as either a single dose ranging from 1 to 600 mg or as four consecutive doses of 150 mg administered every other week. As secondary objectives, the PK and PD (i.e., inhibition of capsaicin-induced DBF) of galcanezumab were evaluated. Over the dose range tested, galcanezumab exposure increased in proportion to dose, signifying linear PK. In addition, a robust, durable, and dose-dependent inhibition of capsaicin-induced DBF was observed. Consequently, the clinical development of galcanezumab was continued as a prophylactic therapy for migraine.

Galcanezumab is a fully humanized monoclonal antibody that potently and selectively binds to CGRP. Monoclonal antibodies are particularly attractive tools for preventative treatment because they exhibit a lack of off-target toxicity, a long elimination half-life, and clearance by proteolysis. Because galcanezumab is not metabolized by liver enzymes, drug–drug interactions are very unlikely. Taken together, these characteristics should translate into a favorable tolerability profile and patient adherence, and with few contraindications for the intended indication of migraine prevention.

With respect to safety and tolerability of galcanezumab, there was no dose-dependent difference in either type or frequency of treatment-emergent AEs and no clinically meaningful difference when compared with placebo. Although one subject developed liver function abnormalities in the single-dose part of the study, it was likely due to the concomitant intake of ciprofloxacin, prescribed together with paracetamol and ibuprofen. Acute cholestatic hepatitis, as observed in this subject, is an uncommon condition which has previously been reported as a rare idiosyncratic response to ciprofloxacin. In this case, galcanezumab had been administered almost 3 months prior to the incident at a very low dose (5 mg), which, after three elimination half-lives, was almost completely cleared from the body. This case was therefore considered to be related to ciprofloxacin which was supported by the subject's complete recovery after stopping the antibiotic. So far, there is no indication for galcanezumab, or for any of the monoclonal antibodies interfering with the CGRP pathway, to cause hepatotoxicity. Therefore, inhibition of this pathway does not seem to be directly linked to hepatotoxicity.

Another possible safety concern is the potential but unclear role of CGRP as a vasodilator in maintaining cardiovascular homeostasis, raising concerns around the cardiovascular safety of compounds interfering with the CGRP pathway (MaassenVanDenBrink et al., 2016). Because of this, in addition to the traditional cardiovascular safety parameters (i.e., blood pressure, heart rate, and ECG), PWA was included in this study as an exploratory safety parameter to evaluate the impact of galcanezumab on changes in arterial stiffness. Based on PWA, galcanezumab did not affect resting vascular tone. This observation is consistent with earlier findings suggesting that inhibition of the effects of CGRP by blocking the receptor can potently inhibit CGRP-dependent cutaneous vasodilation induced by capsaicin without affecting arterial stiffness under basal conditions (Petersen et al., 2005a; Van der Schueren et al., 2008, 2011). During the course of both parts of the study, there were no notable dose-related trends or changes in ECG parameters relative to predose or placebo. Vital signs data did not reveal any safety concerns, and no relevant cardiovascular AEs related to galcanezumab were reported. These findings are consistent with non-clinical and clinical experience with CGRP antagonists so far and continue to support the cardiovascular safety of these compounds (Iovino et al., 2004; Petersen et al., 2005a,b; Zeller et al., 2008). This is in contrast to current therapies used for the treatment of migraine headache, in particular the ergot alkaloids and the triptans, which both increase blood pressure and arterial stiffness and are therefore formally contraindicated in patients with a higher risk for cerebrovascular or cardiovascular events (de Hoon et al., 2000, 2001; Vanmolkot and de Hoon, 2006; Depré et al., 2013).

The safety of galcanezumab is further supported by more recent data collected in a Phase II study of 218 migraine patients, 108 of whom received 150 mg of galcanezumab every 2 weeks for 12 weeks (Dodick et al., 2014b). The results of that study over longer repeated exposure to galcanezumab demonstrated no clinically relevant drug-related changes in any safety parameters. In more general terms, ongoing studies with the various monoclonal antibodies directed against CGRP as a ligand (i.e., galcanezumab, eptinezumab, and fremanezumab), as well as against the CGRP receptor (erenumab), are building evidence of both hepatic and cardiovascular safety (Iovino et al., 2004; Olesen et al., 2004; Petersen et al., 2005a,b; Van der Schueren et al., 2011).

A secondary objective of the present study was aimed at establishing a maximal well-tolerated, safe dose suitable for testing in a Phase IIa proof-of-concept study. While a maximum tolerated dose was not determined, the maximum feasible or practical dose was determined. Further, the long terminal elimination half-life of galcanezumab of over 30 days is characteristic for mAbs and suitable for its intended use as a prophylactic treatment for migraine.

The capsaicin-induced DBF assay was integrated as a pharmacodynamic biomarker to facilitate dose selection. Given the pivotal role of CGRP in the increase in DBF following the local application of capsaicin to the skin, inhibition of this response has been used previously to provide evidence of proof of mechanism for CGRP-blocking therapeutics. In this study, galcanezumab showed a robust and durable inhibition of capsaicin-induced DBF after both single and repeated injections. Based on extensive modeling of the PK-PD relationship, a Q2W (i.e., every other week) dose of 150 mg of galcanezumab was selected for the Phase II proof-of-concept study, which confirmed the prophylactic efficacy of galcanezumab in migraine (Vermeersch et al., 2015).

In conclusion, based on all available data from this Phase I study demonstrating safety, tolerability, an attractive PK profile, and proof of mechanism, further development of galcanezumab for the prevention of migraine is warranted.

## Author contributions

DM and JdH contributed to the conception, design, and acquisition of data for this work, and contributed to the drafting of the manuscript and to the critical revision of the manuscript for important intellectual content. EC: contributed to the conception, design, interpretation of data for this work, and has contributed to the critical revision of the manuscript for important intellectual content. CV and AVH contributed to the acquisition and interpretation of data for this work, and contributed to the critical revision of the manuscript for important intellectual content. ER, JS, DG, and TS: contributed to the design and interpretation of data for this work, and contributed to the critical revision of the manuscript for important intellectual content.

## Other information

The trial is registered at ClinicalTrials.gov (NCT 01337596).

### Conflict of interest statement

ER and EC are employees of Eli Lilly and Company. DM and JS were employees of Eli Lilly and Company at the time the study was conducted. JdH acted as Principal Investigator, for which the university received research grants from Abide, Amgen, Galderma, Genentech, GlaxoSmithKline, Janssen Research & Development, Lilly Chorus, MSD, Novartis, Sanofi Pasteur, UCB, and Vertex; and acted as a consultant for Ablynx, Amgen, Eli Lilly, Genentech, and UCB. CV acted as Principal Investigator, for which the University received research grants from AdImmune, GlaxoSmithKline, and Sanofi Pasteur. DG and TS were employees of Arteaus Therapeutics at the time of the study. AV declare that the research was conducted in the absence of any commercial or financial relationships that could be construed as a potential conflict of interest.
